# The Transition to Family Caregiving: Does It Affect Biomarkers of Aging?

**DOI:** 10.1093/geroni/igaa057.2275

**Published:** 2020-12-16

**Authors:** Shang-En Chung, David Roth, John Bentley, Jeremy Walston

**Affiliations:** 1 Johns Hopkins University, Baltimore, Maryland, United States; 2 University of Mississippi, Oxford, Mississippi, United States

## Abstract

Blood samples were collected from participants in the REGARDS study on two separate occasions. No participants in the Caregiving Transitions Study were caregivers at the first blood draw, but 251 became caregivers before the second blood draw 9 years later. These caregivers were matched with 251 noncaregiving controls. Six circulating biomarkers of inflammation (e.g., CRP, IL-6, TNFR1) and a measure of cellular aging (leukocyte telomere length) were assessed at both blood draws. All biomarkers except CRP showed overall aging effects (ps < 0.001). Caregivers had a small but significantly greater increase in TNFR1 levels (p = 0.03) than controls, but no significant differential changes were found on the other 5 inflammatory biomarkers or on telomere length. Preliminary findings from latent variable models indicated good model fit and found caregivers to be 0.27 SDs lower than controls on a latent construct of inhibitory, regulatory feedback of systemic inflammation (p = 0.03).

